# Divergent pathway of lipid profile components for cardiovascular disease and mortality events: Results of over a decade follow-up among Iranian population

**DOI:** 10.1186/s12986-016-0102-1

**Published:** 2016-06-24

**Authors:** Zahra Ghasemzadeh, Hengameh Abdi, Samaneh Asgari, Maryam Tohidi, Davood Khalili, Majid Valizadeh, Siamak Moeini, Vahid Eidkhani, Fereidoun Azizi, Farzad Hadaegh

**Affiliations:** Prevention of Metabolic Disorders Research Center, Research Institute for Endocrine Sciences, Shahid Beheshti University of Medical Sciences, P.O. Box: 19395-476, Tehran, Iran; Endocrine Research Center, Research Institute for Endocrine Sciences, Shahid Beheshti University of Medical Sciences, Tehran, Iran; Obesity Research Center, Research Institute for Endocrine Science, Shahid Beheshti University of Medical Sciences, Tehran, Iran

**Keywords:** Cardiovascular disease, Mortality, Lipids, Cholesterol, Triglycerides

## Abstract

**Background:**

Data regarding the impact of different lipid measures on cardiovascular diseases (CVD) and mortality events is not consistent. We aimed to evaluate the relationship between different lipid parameters and incident CVD and mortality events in an Iranian population over a median follow-up of 11.9 years.

**Methods:**

The study was conducted on 2532 men and 2986 women aged ≥ 40 years. Multivariate adjusted hazard ratios (HRs), using age as time scale, were calculated for every 1 standard deviation (SD) increase in total cholesterol (TC), logarithm-transformed triglycerides (ln-TGs), low density lipoprotein-cholesterol (LDL-C), high density lipoprotein-cholesterol (HDL-C), non-HDL-C, TC/HDL-C and ln-TGs/HDL-C. Covariates included gender (female as reference), body mass index, education status, low physical activity, smoking, blood pressure status (normotension, prehypertension and hypertension), glucose tolerance status (normal glucose tolerance, prediabetes and diabetes) and lipid lowering drugs. The same analyses were also repeated for tertiles of all lipid measures. Considering the absence of interaction between gender and lipid parameters, we used a sex-adjusted analysis. For analyses of mortality events, prevalent CVD was adjusted as well (All p for interactions > 0.1).

**Results:**

A total of 789 new CVD events, 279 cardiovascular (CV) and 270 non-CV deaths occurred. In multivariate analysis, all lipid measures except HDL-C showed significant risk for new CVD events with HRs ranged from 1.14 to 1.27 for ln-TGs/HDL-C and LDL-C, respectively (all *p*-values ≤ 0.001). Considering CV mortality, there were significant positive associations between TC, LDL-C, non-HDL-C, TC/HDL-C and CV mortality events in sex-adjusted analysis; however after multivariate analysis, these associations attenuated and reached to null. Applying lipid measures as categorical variables, only TC displayed a positive association with CV mortality in multivariate analysis [TC ≥ 6.14 mmol/L: HR 1.43 (1.04–1.98)].

In multivariate analysis, there were negative significant associations between all lipid measures except HDL-C and non-CV mortality; every 1-SD increase in TC, LDL-C, non-HDL-C, ln-TGs ,TC/HDL-C and ln-TGs/HDL-C was associated with 24, 25, 27, 19, 23 and 17 % decreased risk in non-CV mortality (all *p*-values ≤ 0.01).

**Conclusions:**

These findings indicate divergent associations of TC, LDL-C, non-HDL-C, TC/HDL-C, TGs and TGs/HDL-C with CVD vs non-CV mortality, demonstrating a higher risk for the former and lower risk for the latter.

**Electronic supplementary material:**

The online version of this article (doi:10.1186/s12986-016-0102-1) contains supplementary material, which is available to authorized users.

## Background

Cardiovascular disease (CVD) is the leading cause of 30 % of mortality worldwide [1] and up to 50 % of deaths in Iran [2]. The association between different lipid measures and cardiovascular disease and mortality have been shown in several studies [3–5]. Based on the results of observational and interventional studies conducted in middle-aged populations, there is a continuous graded relationship between serum total cholesterol (TC) levels with CVD and mortality [6]. Meanwhile, non-high density lipoprotein cholesterol (non-HDL-C) as the masses of cholesterol in the atherogenic apo B lipoprotein particles has been known as a valuable predictor for cardiovascular risk [3]. In a cohort of Western population, triglycerides (TGs) and the TC to HDL-C ratio (TC/HDL-C) were more strongly associated with risk of future coronary heart disease (CHD) [7]. Moreover, a recent meta-analysis showed significant association between serum triglycerides (TGs) and CVD and all-cause mortality [5]. There are several studies regarding inverse relationship between HDL-C levels and CVD [8]. Furthermore, many studies have shown that indices such as TGs to HDL-C ratio (TG/HDL-C) and TC/HDL-C can also predict CVD and CHD mortality [4, 9–11]. Despite these, data regarding association between lipid measures and mortality is not consistent [12, 13] and to the best of our knowledge, few studies have examined this relationship considering cardiovascular (CV) vs non-CV mortality, separately.

Almost all previous surveys regarding the association between lipid measures and CVD or all-cause mortality events have been performed on European, American or the Far East populations [14, 15] and it is not clear whether their results can be extrapolated to Middle-Eastern population who have high incidence of CHD and mortality events [16, 17]. Recently, a multivariate sex-adjusted analysis showed that among Iranian adults aged ≥ 50 years, serum levels of TC, TGs, low density lipoprotein-cholesterol (LDL-C), non-HDL-C, and TC/HDL-C were significantly associated with higher risk of incident CHD, whereas HDL-C was associated with a lower risk [18].

In the current population-based study, we expand our previous findings and investigate the association between seven lipoprotein measures and CVD events, CV and non-CV mortality in more than a decade follow-up.

## Methods

### Study population

Subjects for the current study were selected from participants of the Tehran Lipid and Glucose Study (TLGS), a long term prospective population-based study being conducted on a representative sample of Tehranian residents to determine the prevalence and incidence of non-communicable diseases and related risk factors. Details of TLGS have been published previously [19]. TLGS has two main phases: a cross-sectional phase (1999–2001) and a prospective ongoing phase consisting of follow-up visits at 3-year intervals.

A total of 6445 individuals, aged ≥ 40 years were enrolled in the first (*n* = 5406) or second (*n* = 1039) phases. To evaluate the effects of lipid markers on CVD and non-CV mortality, after excluding participants without any follow-up (*n* = 618) or with missing data for baseline covariates (*n* = 309), 5518 participants with complete data were followed for a median of 11.9 years (interquartile range: 7.8–13.1 years) up to March 2012. For investigating the effects of lipid measures on CVD events, the analysis was done in a smaller sample (*n* = 5054) by excluding individuals with prevalent CVD.

During the third phase of the TLGS (2006–2008), a total of 4920 participants were randomly selected for completing the dietary assessment based on their age and sex. Finally, the dietary data for 3462 subjects were completed using a validated 168-item food frequency questionnaire [20]. For the present study, after exclusion of participants aged < 40 years and those with under- or over-reporting of dietary energy intakes (<800 or ≥ 4200 kilocalories/day), dietary information of 915 participants was included in a sensitivity analysis. Furthermore, data regarding serum insulin was available for 1548 participants at baseline which was considered in a subgroup analysis.

The ethics committee of the Research Institute for Endocrine Sciences, Shahid Beheshti University of Medical Sciences approved the design of TLGS and all participants provided written informed consent.

### Data collection

#### Medical history, clinical examination and laboratory measurements

Participants were interviewed face to face by trained interviewers. A standard questionnaire was used to collect demographic information, including their smoking status and taking of any antidiabetic or hypertension drugs. Systolic blood pressure (SBP) and diastolic blood pressure (DBP) were measured twice in a seating position on the right arm, using a standard mercury sphygmomanometer and the mean value was considered as the subject’s SBP and DBP.

Weight was measured with individuals minimally clothed without shoes, using digital scales (Seca 707: range 0.1–150 kg) and recorded to the nearest 0.1 kg. Height was measured in a standing position without shoes, using a tape meter, while shoulders were in a normal alignment. Body mass index (BMI) was calculated as weight in kilograms divided by height in square meters. Waist circumference (WC) was measured at the level of umbilicus.

Venous samples were collected in vacationer tubes after 12–14 h overnight fasting between 7:00 and 9:00 A.M and centrifuged within 30–45 min of collection. Fasting plasma glucose (FPG) and 2-h post challenge plasma glucose (2 h-PCPG) were measured by the enzymatic colorimetric method, using glucose oxidase; inter- and intra-assay coefficients of variation at baseline and follow-up phases were both less than 2.3 %. TC was assayed, using the enzymatic colorimetric method with cholesterol esterase and cholesterol oxidase. HDL-C was measured after precipitation of the apolipoprotein B (apo B)-containing lipoproteins with phosphotungistic acid. TGs were assayed using glycerol phosphate oxidase. Both intra- and inter-assay coefficients of variations for TC, HDL-C and TGs were less than 1.9, 3 and 2.1 %, respectively. Analyses were performed using related kits (Pars Azmon Inc., Tehran, Iran) and a Selecta 2 auto-analyzer (Vital Scientific, Spankeren, Netherlands) on the day of blood sampling. All assays were done when quality control met the acceptable criteria. To calculate LDL-C, a modified Friedewald equation was used [21].

### Definition of terms

Participants were classified as having type 2 diabetes if they met at least one of the following criteria: FPG ≥ 7 mmol/L, 2 h-PCPG ≥ 11.1 mmol/L or taking antidiabetic medications. Moreover, prediabetes was defined as having a 5.55 mmol/L ≤ FPG < 7 mmol/L and/or a 7.77 mmol/L ≤ 2 h-PCPG < 11.1 mmol/L, without using glucose lowering drugs; those with FPG < 5.55 mmol/L and 2 h-PCPG < 7.77 mmol/L were considered as normal glucose tolerant according to the definition of American Diabetes Association [22]. Hypertension was defined as systolic blood pressure (SBP) ≥ 140 mmHg or diastolic blood pressure (DBP) ≥ 90 mmHg, or taking antihypertensive medications. Prehypertension was defined as SBP ≥ 120 mmHg and < 140 mmHg and DBP ≥ 80 mmHg and < 90 mmHg and normotension as SBP < 120 mmHg and DBP < 80 mmHg without any medication use [23]. A current smoker was defined as a person who smokes cigarettes daily or occasionally. Low physical activity was defined as vigorous activity less than three times per week for participants entered in first phase and as metabolic equivalent task-minutes per week < 600 for those who entered in the second phase [24]. Education status was categorized into 3 groups: < 6 years, 6–12 years and ≥ 12 years. With respect to the Kidney Disease Outcome Quality Initiative guidelines, chronic kidney disease (CKD) was defined as either kidney damage or estimated Glomerular Filtration Rate (eGFR) < 60 mL/min/1.73 m^2^ for > 3 months [25].

### Outcome measurement

Details of cardiovascular outcomes have been published previously [19]. In the ongoing TLGS, all participants are followed up for any medical event during the previous year by telephone. They are questioned by a trained nurse regarding any medical conditions or whether a related event had occurred; a trained physician collected complementary data during a home visit and/or a visit to the respective hospital to collect data from medical files; in case of mortality, data are collected from the hospital or death certificate by an authorized local physician, then evaluated by an outcome committee consisting of a principal investigator, an internist, an endocrinologist, a cardiologist, an epidemiologist and the physician who collects the outcome data. Other experts are invited for evaluation of non-communicable diseases, as needed. A specific outcome for each event is assigned, based on the International Statistical Classification of Diseases and Related Health Problems criteria, 10th Revision, and the American Heart Association classification for cardiovascular events [19, 26]. In this study, first fatal and non-fatal CHD event, stroke or cerebrovascular accidents were considered CVD events. By definition, CHD includes cases of definite myocardial infarction diagnosed by electrocardiogram (ECG) and biomarkers, probable myocardial infarction (positive ECG findings plus cardiac symptoms or signs and biomarkers showing negative or equivocal results), unstable angina pectoris (new cardiac symptoms or changing symptom patterns and positive ECG findings with normal biomarkers) and angiographic proven CHD. CV mortality is specified as a composite measure of any fatal CVD events, including fatal CHD and fatal stroke.

### Statistical analysis

Mean and standard deviation (SD) values for continuous and frequencies (%) for categorical variables of the baseline characteristics were described and median (interquartile range) for TGs and TGs/HDL-C as they had skewed distribution.

Cox proportional hazard models with age as time scale were used to evaluate associations of CVD events, CV and non-CV mortality with one standard deviation (1-SD) change in TC, LDL-C, HDL-C, non-HDL-C as well as 1-SD increase in TC/HDL-C [27]. For TGs and TGs/HDL-C, hazard ratios (HRs) were calculated for every 1-SD increase in logarithm (ln)-transformed form of these measures. The event time was defined as the time between entrance to the study and the endpoints. Endpoints were considered as CVD event, CV and non-CV mortality. Also censored data was defined as individuals either lost to follow-up, having left residential area, having non-CV mortality (for CV mortality endpoint) or continued up to March 2012, whichever occurred earlier.

Variables with *p*-values < 0.2 in univariate analysis were included in the multivariate Cox model. Interaction between gender and lipid measures with CVD event, CV and non-CV mortality was checked by log–likelihood ratio test, in multivariate analysis; since, there was no significant interaction (all *p*-values > 0.1), analysis was repeated in a pooled sample to reach acceptable statistical power. Also, the effect of lipid measures on CVD events was analysed in both genders. The interaction between prevalent CVD and lipid lowering drugs with lipid markers was checked and since no significant interactions were found (all *p*-values > 0.1), they were adjusted in multivariate model.

In multivariate analysis, two models were applied; model 1 included baseline levels of lipid profiles and gender (female as reference); model 2, further adjusted for potential covariates, included BMI, education status (≥12 years as reference group), low physical activity, smoking, blood pressure status (normotension as reference, prehypertension, hypertension), glucose tolerance status (normal glucose tolerance as reference, prediabetes, diabetes), lipid lowering drugs, and prevalent CVD (for CV and non-CV mortality events). Additionally, analyses were repeated for tertiles of all lipid measures with the same approach. The annual CV and non-CV mortality rate (per 1000 person-year) for each tertile of lipid-measures was calculated by dividing the total number of incident events to the total person-years. The proportionality of the multivariable Cox model was assessed using Schoenfeld’s global test of residuals (*p* > 0.1).

Dietary intakes of nutrients were adjusted for total energy intakes, according to residuals method [28]. Potential confounding effects of homeostatic model assessment-insulin resistance (HOMA-IR) and dietary factors including total fat, dietary cholesterol, total fiber, and Na/K ratio were assessed for sensitivity analysis.

Statistical analyses were performed using STATA version 12.0 (Stata Corp LP, College Station, Texas). *P* values < 0.05 were considered statistically significant.

## Results

The study sample consists of 5518 individuals (2532 men and 2986 women), with mean age (SD) of 55.46 (10.73) years in men and 53.18 (9.36) years in women. Baseline characteristics in men and women are shown in Table 1. There were significant difference in all variables between men and women, except for TGs. Men were older and had lower BMI, TC, LDL-C, HDL-C as well as lower prevalence of prediabetes, diabetes, hypertension and CKD; however, the levels of non-HDL-C, TC/HDL-C, ln-TGs/HDL-C, prevalence of CVD, rate of current smoking, prehypertension and low levels of physical activity were higher in men.

**Table 1:** Baseline characteristics of participants in men, women and total population; Tehran Lipid and Glucose Study (TLGS) (2001–2012)

	Total (*N* = 5518)	Men (*N* = 2532)	Women (*N* = 2986)	*p*-value
Age, years	54.23(10.07)	55.46(10.73)	53.18(9.36)	<0.001
BMI, kg/m2	27.86(4.59)	26.24(3.89)	29.24(4.69)	<0.001
WC, cm	92.78(11.17)	91.26(10.5)	93.46(11.42)	<0.001
SBP, mm Hg	126.78(21.16)	125.96(20.8)	127.47(21.45)	0.008
DBP, mm Hg	80.13(11.49)	79.35(11.88)	80.8(11.10)	<0.001
FPG, mmol/L	5.89(2.22)	5.77(1.96)	5.99(2.41)	<0.001
TC, mmol/L	5.73(1.22)	5.45(1.10)	5.98(1.26)	<0.001
LDL-C, mmol/ L	3.97(1.02)	3.97(0.93)	4.12(1.07)	<0.001
HDL-C, mmol/ L	1.08(0.28)	0.99(0.25)	1.15(0.29)	<0.001
Non-HDL-C, mmol/ L	4.46(1.20)	4.46(1.10)	4.2(1.25)	<0.001
TGs, mmol/ L	1.87(1.33)	1.84(1.35)	1.90(1.31)	0.06
TC/HDL-C	5.63(1.77)	5.82(1.83)	5.46(1.70)	<0.001
TGs/HDL-C	1.81(1.67)	1.95(1.85)	1.70(1.50)	<0.001
Current smoker, *n* (%)	862(15.6)	727(28.7)	135(4.5)	<0.001
Low physical activity, *n* (%)	4401(79.8)	2082(82.2)	2319(77.7)	<0.001
Education level, *n* (%)				<0.001
≥ 12 years	490(8.9)	368(14.6)	122(4.1)	
6-11 years	1939(35.2)	1065(42.1)	874(29.3)	
< 6 years	3082(55.9)	1096(43.3)	1986(66.6)	
Prevalent CVD, *n* (%)	464(8.4)	252(10.0)	212(7.1)	<0.001
Prevalent CKD, *n* (%)	1617(29.3)	494(19.5)	1123(37.6)	<0.001
Glucose tolerance, *n* (%)				0.003
Normal glucose tolerance	3022(57.3)	1442(59.5)	1580(55.4)	
Prediabetes	1445(27.4)	647(26.7)	798(28.0)	
Diabetes	806(15.3)	333(13.7)	473(16.6)	
Blood pressure status, *n* (%)				<0.001
Normotension	1714(31.1)	862(34.0)	852(28.5)	
Prehypertension	1832(33.2)	862(34.0)	970(32.5)	
Hypertension	1972(35.7)	808(31.9)	1164(39.0)	
Hypertension drug, *n* (%)	769(13.9)	230(9.1)	539(18.1)	<0.001
Lipid lowering drugs, *n* (%)	306(5.5)	83(3.3)	223(7.5)	<0.001
Diabetes drug, *n* (%)	424(7.7)	152(6.0)	272(9.1)	<0.001

Data are shown as mean ± SD for continuous variables (*p* value calculated with the t test), *n* (%) for categorical variables (*p*-value according to the chi-squared test) or median (interquartile range) for TGs and TGs/HDL-C (*p*-value according to the Mann–Whitney U test)

*BMI* body mass index, *WC* waist circumference, *SBP* systolic blood pressure, *DBP* diastolic blood pressure, *FPG* fasting plasma glucose, *TC* total cholesterol, *LDL-C* low density lipoprotein cholesterol, *HDL-C* high density lipoprotein cholesterol, *TGs* triglycerides, *CVD* cardiovascular disease, *CKD* chronic kidney disease

We ascertained 789 incident CVD events among participants free of CVD at baseline (*n* = 5054). The incidence of CVD event for each 1000 person-year is shown in Additional file 1: Table S1. In total population, the multivariate adjusted HRs of CVD events for every 1-SD increase in TC, LDL-C, non-HDL-C, ln-TGs, TC/HDL-C and ln-TGs/HDL-C were associated with 26, 27, 22, 15, 18 and 14 %, respectively (Table 2); however, the corresponding change in HDL-C was associated with 7 % decreased risk of CVD, which did not reach to statistical significance (*p* = 0.07). Sex-stratified analysis did not change the association between lipid parameters and CVD events. Analysis using tertiles of lipid measures for incident CVD showed the same results (Table 3).

**Table 2:** Hazard ratios of lipid measures for predicting first cardiovascular disease events among participants without prevalent CVD (*n* = 5054); Tehran Lipid and Glucose Study (TLGS) (2001–2012)^a^

	Model 1			Model 2	
	SD (mmol/L)	HR (95 % CI)	*p*-value	HR (95 % CI)	*p*-value
Men					
TC	1.10	1.34(1.22-1.47)	<0.001	1.3(1.18-1.43)	<0.001
LDL-C	0.93	1.37(1.25-1.51)	<0.001	1.33(1.2-1.46)	<0.001
HDL-C	0.25	0.88(0.79-0.98)	0.021	0.94(0.84-1.04)	0.24
Non-HDL-C	1.11	1.37(1.25-1.5)	<0.001	1.32(1.20-1.45)	<0.001
ln-TGs	0.57	1.20(1.09-1.3)	<0.001	1.1(1.0-1.21)	0.05
TC/HDL-C	1.85	1.10(1.13-1.28)	<0.001	1.17(1.09-1.25)	<0.001
ln-TGs/HDL-C	0.7	1.2(1.1-1.31)	<0.001	1.1(1.0-1.21)	0.056
Women					
TC	1.24	1.30(1.18-1.43)	<0.001	1.21(1.1-1.34)	<0.001
LDL-C	1.05	1.30(1.19-1.43)	<0.001	1.22(1.11-1.35)	<0.001
HDL-C	0.29	0.87(0.78-0.98)	0.02	0.91(0.82-1.02)	0.11
Non-HDL-C	1.23	1.32(1.20-1.45)	<0.001	1.23(1.12-1.36)	<0.001
ln-TGs	0.51	1.42(1.27-1.58)	<0.001	1.24(1.1-1.4)	<0.001
TC/HDL-C	1.69	1.33(1.21-1.46)	<0.001	1.26(1.13-1.4)	<0.001
ln-TGs/HDL-C	0.65	1.38(1.23-1.54)	<0.001	1.22(1.09-1.38)	0.001
Total					
TC	1.21	1.33(1.24-1.42)	<0.001	1.26(1.18-1.35)	<0.001
LDL-C	1.01	1.34(1.26-1.43)	<0.001	1.27(1.19-1.36)	<0.001
HDL-C	0.28	0.88(0.81-0.95)	0.001	0.93(0.86-1.007)	0.07
Non-HDL-C	1.19	1.35(1.26-1.44)	<0.001	1.22(1.14-1.30)	<0.001
ln-TGs	0.53	1.28(1.20-1.37)	<0.001	1.15(1.07-1.24)	<0.001
TC/HDL-C	1.78	1.24(1.18-1.30)	<0.001	1.18(1.12-1.25)	<0.001
ln-TGs/HDL-C	0.67	1.27(1.18-1.36)	<0.001	1.14(1.06-1.23)	<0.001

Model 1: Lipid profile + gender (for total population); model 2 = model 1 + blood pressure status (i.e. normotension, prehypertension and hypertension status), glucose tolerance status (normal glucose tolerance, prediabetes and diabetes), education status, low physical activity, current smoker, lipid lowering drugs and body mass index

^a^Hazard ratios (HR) indicate the increased risk for every 1-SD increase of each lipid parameter

*CVD* cardiovascular disease, *SD* standard deviation, *TC* total cholesterol, *LDL-C* low density lipoprotein cholesterol, *HDL-C* high density lipoprotein cholesterol, *ln-TGs* logarithm-transformed triglycerides, *CI* confidence interval

**Table 3:** Hazard ratios for predicting first cardiovascular disease based on to lipid profile tertiles of participants without prevalent CVD (*n* = 5054); Tehran Lipid and Glucose Study (TLGS) (2001–2012)

	Model 1			Model 2			
	Tertiles of variables			Tertiles of variables			P _for trend*_
	1	2	3	1	2	3	
Men							
TC, mmol/L	Reference	1.28(1.02-1.6)	1.96(1.56-2.46)	Reference	1.28(1.02-1.61)	1.84(1.46-2.32)	<0.001
LDL-C, mmol/L	Reference	1.36(1.08-1.71)	1.88(1.49-2.36)	Reference	1.38(1.09-1.74)	1.76(1.39-2.23)	<0.001
HDL-C, mmol/L	Reference	0.90(0.72-1.12)	0.81(0.64-1.02)	Reference	0.94(0.75-1.17)	0.92(0.72-1.31)	0.44
Non-HDL-C, mmol/L	Reference	1.35 (1.07-1.7)	1.86 (1.48-2.33)	Reference	1.35 (1.07-1.70)	1.72 (1.36-2.18)	<0.001
TGs, mmol/L	Reference	1.24(0.98-1.57)	1.55(1.24-1.95)	Reference	1.1(0.87-1.4)	1.25(0.98-1.60)	0.07
TC/HDL-C	Reference	1.35(1.05-1.73)	1.74(1.37-2.21)	Reference	1.25(0.97-1.61)	1.54(1.2-1.98)	0.001
TGs/HDL-C	Reference	1.32(1.03-1.68)	1.63(1.29-2.06)	Reference	1.20(0.93-1.54)	1.34(1.04-1.72)	0.02
Women							
TC, mmol/L	Reference	1.72(1.18-2.52)	2.23(1.57-3.18)	Reference	1.64(1.20-2.40)	1.98(1.39-2.83)	<0.001
LDL-C, mmol/L	Reference	1.89(1.32-2.72)	2.36(1.67-3.32)	Reference	1.79(1.25-2.58)	2.09(1.48-2.96)	<0.001
HDL-C, mmol/L	Reference	1.14(0.84-1.54)	0.87(0.66-1.14)	Reference	1.19(0.88-1.61)	0.95(0.72-1.26)	0.49
Non-HDL-C, mmol/L	Reference	1.71 (1.19-2.45)	2.39 (1.7-3.36)	Reference	1.57 (1.09-2.27)	2.09 (1.48-2.95)	<0.001
TGs, mmol/L	Reference	1.39(1.02-1.89)	2.09(1.57-2.79)	Reference	1.17(0.85-1.60)	1.5(1.1-2.03)	0.006
TC/HDL-C	Reference	1.56(1.17-2.07)	2.08(1.57-2.74)	Reference	1.39(1.04-1.86)	1.78(1.34-2.36)	<0.001
TGs/HDL-C	Reference	1.45(1.1-1.91)	1.75(1.33-2.31)	Reference	1.23(0.93-1.62)	1.33(1.0-1.77)	0.05
Total							
TC, mmol/L	Reference	1.39(1.15-1.68)	2.02(1.68-2.43)	Reference	1.36(1.12-1.66)	1.85(1.53-2.23)	<0.001
LDL-C, mmol/L	Reference	1.5(1.23-1.81)	2.01(1.67-2.42)	Reference	1.48(1.22-1.79)	1.84(1.52-2.22)	<0.001
HDL-C, mmol/L	Reference	0.97(0.82-1.16)	0.82(0.69-0.97)	Reference	1.01(0.85-1.21)	0.92(0.77-1.09)	0.34
Non-HDL-C	Reference	1.44(1.19-1.75)	2.03(1.69-2.44)	Reference	1.39(1.15-1.70)	1.82(1.51-2.20)	<0.001
TGs, mmol/L	Reference	1.29(1.07-1.55)	1.77(1.48-2.11)	Reference	1.12(0.92-1.35)	1.35(1.12-1.63)	0.001
TC/HDL-C	Reference	1.44(1.19-1.73)	1.89(1.58-2.26)	Reference	1.31(1.08-1.58)	1.63(1.35-1.96)	<0.001
TGs/HDL-C	Reference	1.37(1.15-1.65)	1.69(1.42-2.02)	Reference	1.21(1.001-1.45)	1.33(1.11-1.61)	0.003

Model 1: lipid profile tertiles + gender (for total population); model 2 = model 1 + blood pressure status (i.e. normotension, prehypertension and hypertension status), glucose tolerance status (normal glucose tolerance, prediabetes and diabetes), education status, low physical activity, current smoker, lipid lowering drugs and body mass index

*CVD* cardiovascular disease, *TC* total cholesterol, *LDL-C* low density lipoprotein cholesterol, *HDL-C* high density lipoprotein cholesterol, *TGs* triglycerides, *CI* confidence interval. *P-values were calculated using age scale Cox proportional hazards regression models

During the study period, among the whole population, 549 mortality events (341 men and 208 women) comprising 279 CV mortality (183 men and 96 women) and 270 non-CV mortality (158 men and 112 women) events, occurred. Different causes of non-CV mortality included cancer (*n* = 85), sepsis (*n* = 46), accidents (*n* = 16), chronic obstructive pulmonary diseases (*n* = 2), and unknown or miscellaneous causes (*n* = 121). The incidence of CV and non-CV mortality for each 1000 person-year is shown in Additional file 1: Tables S2 and S3.

There were significant positive associations between TC, LDL-C, non HDL-C and TC/HDL-C and CV mortality events in sex-adjusted analysis (model 1); however after adjustment with different covariates, these associations attenuated and reached to null (Table 4). Applying lipid measures as categorical rather than continuous variables, TC ≥ 6.14 mmol/L was associated with 52 % and 43 % increase in CV mortality risk in model 1 and 2, respectively (Table 5). As shown in Table 6, in sex- and multivariate adjusted analyses, there were negative significant associations between all lipid measures except HDL-C and non-CV mortality so that every 1-SD increase in TC, LDL-C, non-HDL-C, ln-TGs, TC/HDL-C and ln-TGs/HDL-C were associated with 24, 25, 27, 19, 23 and 17 % decrease in non-CV mortality. When lipid measures were applied as categorical variables, the results generally remained unchanged (Table 7). In a sensitivity analysis, excluding individuals who died within 3 years after initiation of the study as well as those with BMI ≤ 18.5 kg/m^2^ (*n* = 164), the association between lipid parameters and mortality events did not change (Data not shown).

**Table 4:** Hazard ratios of lipid measures for predicting cardiovascular mortality among total participants (*n* = 5518); Tehran Lipid and Glucose Study (TLGS) (2001–2012)^a^

	Model 1			Model 2	
	SD (mmol/L)	HR (95 % CI)	*p*-value	HR (95 % CI)	*p*-value
TC	1.22	1.16(1.03-1.31)	0.02	1.08(0.96-1.21)	0.22
LDL-C	1.01	1.16(0.4-0.67)	0.01	1.08(0.96-1.21)	0.19
HDL-C	0.28	0.96(0.85-1.10)	0.6	1.02(0.9-1.15)	0.77
Non-HDL-C	1.20	1.16(1.03-1.31)	0.01	1.02(0.91-1.15)	0.67
ln-TGs	0.32	1.10(0.98-1.24)	0.11	0.97(0.85-1.10)	0.61
TC/ HDL-C	1.77	1.15(1.03-1.28)	0.01	1.06(0.95-1.20)	0.29
ln-TGs/ HDL-C	0.76	1.10(0.97-1.24)	0.13	0.97(0.85-1.10)	0.61

Model 1: lipid profile + gender; model 2 = model 1 + blood pressure status (i.e. normotension, prehypertension and hypertension status), glucose tolerance status (normal glucose tolerance, prediabetes and diabetes), education status, low physical activity, current smoker, lipid lowering drugs, body mass index and prevalent cardiovascular disease

^a^Hazard ratios (HR) indicate the increased risk for a 1-SD increase of each lipid parameter

*SD* standard deviation, *TC* total cholesterol, *LDL-C* low density lipoprotein cholesterol, *HDL-C* high density lipoprotein cholesterol, *ln-TGs* logarithm-transformed triglycerides, *CI* confidence interval

**Table 5:** Crude and multivariate adjusted hazard ratios of cardiovascular mortality based on lipid profile tertiles of total participants (*n* = 5518); Tehran Lipid and Glucose Study (TLGS) (2001–2012)

	Model 1			Model 2			
	Tertiles of variables			Tertiles of variables			
	1	2	3	1	2	3	P _for trend*_
TC, mmol/L	Reference	1.27(0.93-1.73)	1.52(1.12-2.08)	Reference	1.17(0.84-1.62)	1.43(1.04-1.98)	0.027
LDL-C, mmol/L	Reference	1.27(0.93-1.73)	1.42(1.05-1.93)	Reference	1.19(0.86-1.64)	1.29(0.94-1.78)	0.12
HDL-C, mmol/L	Reference	0.89(0.66-1.20)	0.89(0.67-1.19)	Reference	0.98(0.72-1.34)	1.06(0.78-1.42)	0.71
Non-HDL-C	Reference	1.20(0.89-1.63)	1.17(0.87-1.58)	Reference	1.17 (0.85-1.61)	1.24 (0.9-1.71)	0.19
TGs, mmol/L	Reference	0.94(0.7-1.26)	1.14(0.86-1.52)	Reference	0.83(0.61-1.13)	0.95(0.69-1.3)	0.77
TC/HDL-C	Reference	0.96(0.71-1.30)	1.21(0.91-1.61)	Reference	0.93(0.68-1.27)	1.0(0.74-1.36)	0.95
TGs/HDL-C	Reference	1.23(0.93-1.64)	1.1(0.82-1.5)	Reference	0.96(0.71-1.30)	0.85(0.62-1.18)	0.33

Model 1: lipid profile tertiles + gender; model 2 = model 1 + blood pressure status (i.e. normotension, prehypertension and hypertension status), glucose tolerance status (normal glucose tolerance, prediabetes and diabetes), education status, low physical activity, current smoker, lipid lowering drugs, body mass index and prevalent cardiovascular disease

*TC* total cholesterol, *LDL-C* low density lipoprotein cholesterol, *HDL-C* high density lipoprotein cholesterol, *TGs* triglycerides, *CI* confidence interval

*P-values were calculated using age scale Cox proportional hazards regression models

**Table 6:** Hazard ratios of lipid measures for predicting non-cardiovascular mortality among total participants (*n* = 5518); Tehran Lipid and Glucose Study (TLGS) (2001–2012)^a^

	Model 1			Model 2	
	SD (mmol/L)	HR (95 % CI)	*p*-value	HR (95 % CI)	*p*-value
TC	1.22	0.8(0.70-0.92)	0.001	0.76(0.66-0.87)	<0.001
LDL-C	1.01	0.79(0.69-0.90)	<0.001	0.75(0.66-0.86)	<0.001
HDL-C	0.28	1.08(0.95-1.22)	0.24	1.07(0.94-1.21)	0.32
Non-HDL-C	1.20	0.79(0.69-0.90)	<0.001	0.73(0.64-0.84)	<0.001
ln-TGs	0.32	0.89(0.78-1.0)	0.06	0.81(0.7-0.93)	0.002
TC/HDL-C	1.77	0.81(0.71-0.94)	0.004	0.77(0.67-0.89)	0.001
ln-TGs/HDL-C	0.76	0.89(0.79-1.01)	0.076	0.83(0.72-0.95)	0.006

Model 1: lipid profile + gender; model 2 = model 1 + blood pressure status (i.e. normotension, prehypertension and hypertension status), glucose tolerance status (normal glucose tolerance, prediabetes and diabetes), education status, low physical activity, current smoker, lipid lowering drugs, body mass index and prevalent cardiovascular disease

^a^Hazard ratios indicate the increased risk for a 1-SD increase of each lipid parameter

*SD* standard deviation, *TC* total cholesterol, *LDL-C* low density lipoprotein cholesterol, *HDL-C* high density lipoprotein cholesterol, *ln-TGs* logarithm-transformed triglycerides, *CI* confidence interval

**Table 7:** Crude and multivariate adjusted hazard ratios of non-cardiovascular mortality based on lipid profile tertiles of total participants (*n* = 5518); Tehran Lipid and Glucose Study (TLGS) (2001–2012)

	Model 1			Model 2			
	Tertiles of variables			Tertiles of variables			
	1	2	3	1	2	3	P _for trend*_
TC, mmol/L	Reference	0.73(0.55-0.98)	(0.61-0.45-0.83)	Reference	0.75(0.55-1.01)	0.57(0.42-0.79)	0.001
LDL-C, mmol/L	Reference	0.62(0.46-0.83)	0.61(0.45-0.82)	Reference	0.64(0.47-0.87)	0.57(0.42-0.77)	<0.001
HDL-C, mmol/L	Reference	0.86(0.63-1.19)	1.01(0.75-1.34)	Reference	0.97(0.7-1.34)	1.07(0.79-1.45)	0.62
Non-HDL-C	Reference	0.61(0.45-0.82)	0.63(0.47-0.84)	Reference	0.62(0.46-0.84)	0.58(0.43-0.79)	<0.001
TGs, mmol/L	Reference	0.87(0.66-1.15)	0.71(0.52-0.96)	Reference	0.8(0.60-1.08)	0.59(0.42-0.82)	0.002
TC/HDL-C	Reference	0.78(0.58-1.03)	0.66(0.49-0.90)	Reference	0.77(0.58-1.03)	0.6(0.44-0.83)	0.002
TGs/HDL-C	Reference	0.91(0.69-1.21)	0.79(0.58-1.06)	Reference	0.82(0.61-1.10)	0.65(0.47-0.91)	0.01

Model 1: lipid profile tertiles + gender; model 2 = model 1 + blood pressure status (i.e. normotension, prehypertension and hypertension status), glucose tolerance status (normal glucose tolerance, prediabetes and diabetes), education status, low physical activity, current smoker, lipid lowering drugs, body mass index and prevalent cardiovascular disease

*TC* total cholesterol, *LDL-C* low density lipoprotein cholesterol, *HDL-C* high density lipoprotein cholesterol, *TGs* triglycerides, *CI* confidence interval

*P-values were calculated using age scale Cox proportional hazards regression models

The association between lipid measures and CVD events among 1548 participants, free of prevalent CVD at baseline and available data on HOMA-IR, is shown in Additional file 1: Tables S4 and S5. There was significant association between lipid parameters and CVD events only in sex-adjusted analysis; however, applying lipid measures as categorical variables, significant risk for CVD events was displayed in the second and third tertiles of TC, LDL-C and non-HDL-C also third tertile of TC/HDL-C.

## Discussion

This study determined relationship between different lipid measures with CVD, CV and non-CV mortality during a long term follow-up in an Iranian adult population. Our findings indicate a divergent associations of TC, LDL-C, non-HDL-C, TC/HDL-C, ln-TGs and ln-TGs/HDL-C with CVD vs non-CV mortality, with a higher risk for the former and lower risk for the latter. The absence of any interaction between gender and lipid parameters indicated that being male vs female did not affect the impact of lipid parameters on CVD, CV and non-CV mortality events.

### Lipid measures and cardiovascular events and CV mortality

Cholesterol is a major component of atherosclerosis and CVD as an expression of atherosclerotic processes. There are extensive epidemiological data demonstrating that high blood cholesterol levels increase CVD and CV mortality [6, 11, 15, 29]. In our study, TC ≥ 6.14 mmol/L was associated with 85 and 43 % increase in CVD and CV mortality, respectively. This finding is in agreement with World Health Organization recommendations for CVD risk assessment in low and medium resource countries [30].

In the current study, every 1-SD increment in non-HDL-C and LDL-C was associated with 22 and 27 % increased CVD risk, respectively, findings in line with the results of a meta-analysis regarding markers of cardiovascular risk that demonstrated standardized relative risk for every 1-SD increase in non-HDL-C and LDL-C was 34 and 25 % , respectively [3]. Meanwhile, non-HDL-C ≥ 4.09 mmol/L was associated with 39 % increase in risk of CVD and further 1 mmol/L increment duplicated the risk. In a recent genetic study including data source from TLGS, several variants with large effects on lipid concentrations and their causal relationship with coronary artery disease (CAD) have been discovered; among them, the non-HDL cholesterol genetic risk score has been associated most strongly with CAD [31].

In the present study, increase in serum TGs and TGs/HDL-C was associated with increased CVD events, but not CV mortality. We have previously shown that among Iranian population aged ≥ 50 years, a 1-SD increment in ln-TGs is significantly associated with more than 27 % increased risk for CHD [18]. A long-standing association exists between elevated TG levels and CVD [32–34]. Epidemiological and genetic evidence supporting impact of raised TG remnant cholesterol or triglyceride-rich lipoproteins (TRL) as an additional cause of CVD and all-cause mortality. Nordestgaard et al., acknowledged that TG concentrations of 2–10 mmol/L create an increased risk of CVD [35]. Moreover, mendelian randomization that can contribute to the evidence or against the causal effect of plasma lipids on atherosclerosis hypothesized causality of TG in CAD risk [36]. Several new variants of angiopoietin-like 4, a lipoprotein lipase inhibitor, leading to decreased serum TG levels, have been associated with protection from CVD [37, 38].

There was no significant association between HDL-C and CVD and CV mortality in our study. Different types of HDL-C particles based on their free cholesterol/phospholipid ratio and availability of apolipoprotein A-1 (apo A-1), exhibit different biological effects including reverse cholesterol transport resulting in different levels of cardio-protection [39]. Jensen et al., report that dysfunctional HDL-C containing apo lipoprotein CIII (apo CIII), stimulates inflammatory and atherogenic responses in cells involved in mortality and atherosclerosis [40]. The level of apo CIII is affected by race/ethnicity [41]. Asians have more dysfunctional apo CIII-containing HDL-C than Western population [42], a condition that could predispose them to higher mortality risk with increasing levels of HDL-C. A recent study conducted on TLGS data revealed increasing HDL-C as a CHD risk factor for premenopausal women [43]. Based on the findings from the Lipoprotein Investigators Collaborative study, some HDL-C subclasses may be responsible for the inverse association of HDL-C with CHD [44]. Moreover, a survey by Rohatgi et al., in a population-based cohort, revealed that although baseline HDL-C level was not associated with CVD, cholesterol efflux capacity, as a novel biomarker of reverse cholesterol transport, was inversely associated with the incidence of CVD [45]. On the other hand, large population-based studies have displayed that subjects who are carriers of scavenger receptor BI (the major receptor for HDL-C) mutation have significantly increased levels of plasma HDL-C but an increased risk of CHD (odds ratio = 1.79) [46]. To summarize, for evaluation of the effect of HDL-C on CVD outcomes and mortality events, recent studies emphasize the importance of HDL phenotypes rather than its measured concentration.

### Lipid measures and non-cardiovascular mortality

In the present study, multivariate analysis showed that every 1-SD increment in all lipid measures except HDL-C was associated with 17–27 % decreased risk of non-CV mortality. Several studies have demonstrated the inverse association between lipid parameters and mortality in specific subpopulations including the elderly and those with CKD [13, 47]; however, in the current study the presence of CKD did not remain as a covariate even in the univariate analysis (*P* > 0.6). In a population-based study of individuals aged ≥ 50 years in Denmark, higher lipoprotein levels (including TC and LDL-C) showed a survival benefit compared with recommended low levels [13]. In the Norwegian Counties Study, it was revealed that the second and third quintiles of TGs (1.10 to 1.93 mmol/L) among men were significantly associated with lower risk of all-cause mortality events [48]. Among population of the present study, the inverse relationship between lipid measures and non-CV mortality events did not change after excluding participants with survival < 3 years and those with BMI ≤ 18.5 kg/m^2^ at baseline. The demonstrated inverse association between lipid parameters and non-CV mortality might be due to the residual effect of other conditions such as malnutrition, inflammation and sarcopenia [49, 50] that could not be assessed in our study. Furthermore, socioeconomic status exposing the individuals to a wide range of risk factors for poor health would explain some of the aforementioned inverse relationship for non-CV mortality [51, 52].

### Limitations and strengths

There are several limitations that should be addressed. First, we measured lipid parameters only once at baseline; thus the potential bias resulting from regression dilution could not be ignored. The underestimation of the impact of lipids increased with time interval between baseline and follow-up measurements for TC and TGs, has previously been reported in TLGS cohort [53]. Second, we applied modified Friedwald formula to calculate the level of LDL-C rather than its direct measurement. The collected data are remarkably consistent that LDL particle (LDL-P) number is a better predictor of CV risk compared with the standard measurement of LDL-C concentration; however, these important but costly atherogenic lipid particles were not measured in this large population-based study. On the other hand, still, we need standardization for LDL-P measurements [54]. Third, apo A-1, apo B and lipoprotein(a) (Lp[a]) were not measured in the current study; however, Ingelsson et al. have shown that overall performance of apo B/apo A-1 for prediction of CHD is comparable with that of traditional lipid ratios and results in no incremental utility over TC/HDL-C [55]. Furthermore, a recent meta-analysis including 37 prospective cohorts demonstrated that information on non-HDL-C, apo B, apo A-I and Lp(a) did not improve CVD prediction provided by simple measurements of TC and HDL-C [56]. Although the Emerging Risk Factors Collaboration displayed continuous associations of Lp(a) concentration with risk of CHD, they claimed that there is significant variability in measured Lp(a) concentration [57]. Forth, we did not have data regarding inflammatory parameters including C-reactive protein (CRP) and fibrinogen. Yeboah et al., have reported improvement in the Framingham Risk Score for prediction of CHD within intermediate-risk participants using additional risk markers; nevertheless, the net reclassification improvement for incident CHD with high-sensitivity CRP was less than coronary artery calcium, brachial flow-mediated dilation, ankle-brachial index, carotid intima-media thickness and family history [58]. On the other hand, during a short term follow-up among Tehranian adult population, we have shown that measurement of CRP has no added value for prediction of CVD, over and above traditional risk factors [59]. Fifth, no information about nutrition transition of our country was available in this period; an issue that might affect the impact of lipid measures on mortality outcomes. Sixth, the statistical power of our study to detect a HR of 1.08 of TC (35 %) and LDL-C (25 %) for CV mortality was low. Lastly, this study was performed in a large sample of urban Tehranian population; hence it is not possible to extrapolate our findings to the other parts of country or Middle-Eastern region.

The strength of our prospective study lies in a reasonable sample size, length of follow-up and direct measurement of the different variables and outcomes rather than self-reported data.; moreover, to the best, it was the first long term prospective and population-based study which investigated the association between different lipid measures and CV vs non-CV mortality.

## Conclusions

Findings of the present study indicate divergent associations between TC, LDL-C, non-HDL-C, TC/HDL-C, ln-TGs and ln TGs/HDL-C and CVD vs non-CV mortality, with a higher risk for the former and lower risk for the latter, during a long term population-based study.

## Abbreviations

2 h-PCPG, 2 h- Post Challenge Plasma Glucose; apo A-1, apolipoprotein A-1; apo B, apolipoprotein B; apo CIII, apolipoprotein CIII; BMI, body mass index; CAD, coronary artery disease; CHD, coronary heart disease; CKD, chronic kidney disease; CRP, C-reactive protein; CV, cardiovascular; CVD, cardiovascular disease; DBP, diastolic blood pressure; eGFR, estimated Glomerular Filtration Rate; FPG, fasting plasma glucose; HDL-C, high density lipoprotein-cholesterol; HOMA-IR, homeostatic model assessment-insulin resistance; HR, hazard ratio; LDL-C, low density lipoprotein-cholesterol; ln, logarithm-transformed; Lp(a), lipoprotein(a); SBP, systolic blood pressure; SD, standard deviation; TC, total cholesterol; TGs, triglycerides; TLGS, tehran lipid and glucose study; TRL, triglyceride-rich lipoproteins; WC, waist circumference.

## Additional file

Additional file 1: Table S1. cardiovascular disease event incidence (per 1000 person-year) of different serum lipid markers and lipid indices among men, women and total participants (*n*=5054); Tehran Lipid and Glucose Study (TLGS) (2001-2012). **Table S2** cardiovascular mortality rate (per 1000 person-year) of different serum lipid markers and lipid indices among total participants (*n*=5518); Tehran Lipid and Glucose Study (TLGS) (2001-2012). **Table S3** Non-cardiovascular mortality rate (per 1000 person-year) of different serum lipid markers and lipid indices among total participants (*n*=5518); Tehran Lipid and Glucose Study (TLGS) (2001-2012). **Table S4** Hazard ratios of lipid measures for predicting first cardiovascular disease events among participants without prevalent CVD in those with available data on HOMA-IR (*n*=1548); Tehran Lipid and Glucose Study (TLGS) (2001-2012)*. **Table S5** Hazard ratios of lipid measures for predicting first cardiovascular disease events among participants without prevalent CVD based on lipid profile tertiles CVD in those with available data on HOMA-IR (*n*=1548); Tehran Lipid and Glucose Study (TLGS) (2001-2012). (DOCX 65 kb)
