# Supplementary material for: Divergent pathway of lipid profile components for cardiovascular disease and mortality events: Results of over a decade follow-up among Iranian population
Source: Nutr Metab (Lond). 2016 Jun 24;13:43. doi: 10.1186/s12986-016-0102-1 (PMC4919865; doi:10.1186/s12986-016-0102-1)
Supplement: Additional file 1: Table S1. — cardiovascular disease event incidence (per 1000 person-year) of different serum lipid markers and lipid indices among men, women and total participants (n=5054); Tehran Lipid and Glucose Study (TLGS) (2001-2012). Table S2 cardiovascular mortality rate (per 1000 person-year) of different serum lipid markers and lipid indices among total participants (n=5518); Tehran Lipid and Glucose Study (TLGS) (2001-2012). Table S3 Non-cardiovascular mortality rate (per 1000 person-year) of different serum lipid markers and lipid indices among total participants (n=5518); Tehran Lipid and Glucose Study (TLGS) (2001-2012). Table S4 Hazard ratios of lipid measures for predicting first cardiovascular disease events among participants without prevalent CVD in those with available data on HOMA-IR (n=1548); Tehran Lipid and Glucose Study (TLGS) (2001-2012)*. Table S5 Hazard ratios of lipid measures for predicting first cardiovascular disease events among participants without prevalent CVD based on lipid profile tertiles CVD in those with available data on HOMA-IR (n=1548); Tehran Lipid and Glucose Study (TLGS) (2001-2012). (DOCX 65 kb) [file 12986_2016_102_MOESM1_ESM.docx]

| Supplemental Table S1 cardiovascular disease event incidence (per 1000 person-year) of different serum lipid markers and lipid indices among men, women and total participants (n=5054); Tehran Lipid and Glucose Study (TLGS) (2001-2012). | | | | | | | | | | | |  |
| --- | --- | --- | --- | --- | --- | --- | --- | --- | --- | --- | --- | --- |
|  | |  | | **Lipid limits** | | **Event/At risk** | | **Person-year** | | **CVD incidence^*^**  **(95% CI)** | |  |
| Men (N=458) | |  | |  | |  | |  | |  | |  |
| TC | |  | |  | |  | |  | |  | |  |
|  | T1 | |  | | <5.15 | | 145/965 | | 9744 | | 14.9(12.6-17.5) | |
|  | T2 | |  | | 5.15-6.14 | | 156/774 | | 8316 | | 18.8(16.0-21.9) | |
|  | T3 | |  | | ≥6.14 | | 157/541 | | 5805 | | 27.0(23.1-31.6) | |
| LDL-C | |  | |  | |  | |  | |  | |  |
|  | T1 | |  | | <3.49 | | 135/919 | | 9283 | | 14.5(12.3-14.2) | |
|  | T2 | |  | | 3.49-4.32 | | 159/766 | | 8216 | | 19.3(16.7-22.6) | |
|  | T3 | |  | | ≥4.32 | | 164/595 | | 6365 | | 25.8(22.1-30.0) | |
| HDL-C | |  | |  | |  | |  | |  | |  |
|  | T1 | |  | | <0.93 | | 228/1122 | | 11691 | | 19.5(17.1-22.2) | |
|  | T2 | |  | | 0.93-1.19 | | 124/630 | | 6582 | | 18.8(15.8-22.5) | |
|  | T3 | |  | | ≥1.19 | | 106/528 | | 5591 | | 18.9(15.7-22.9) | |
| Non-HDL-C | |  | |  | |  | |  | |  | |  |
|  | T1 | |  | | <4.09 | | 1.5/899 | | 9085 | | 14.8(12.5-17.6) | |
|  | T2 | |  | | 4.09-5.08 | | 159/773 | | 8287 | | 19.2(16.4-22.4) | |
|  | T3 | |  | | ≥5.08 | | 164/608 | | 6492 | | 25.3(21.7-29.4) | |
| TGs | |  | |  | |  | |  | |  | |  |
|  | T1 | |  | | <1.5 | | 138/797 | | 8078 | | 17.1(14.4-20.2) | |
|  | T2 | |  | | 1.5-2.33 | | 144/723 | | 7616 | | 18.9(16.0-22.2) | |
|  | T3 | |  | | ≥2.33 | | 176/760 | | 8171 | | 21.5(18.6-24.9) | |
| TC/HDL-C | |  | |  | |  | |  | |  | |  |
|  | T1 | |  | | <4.75 | | 103/662 | | 6761 | | 15.2(12.5-18.5) | |
|  | T2 | |  | | 4.75-6.12 | | 153/771 | | 8150 | | 18.8(16.0-22.0) | |
|  | T3 | |  | | ≥6.12 | | 202/847 | | 8953 | | 22.7(19.6-25.9) | |
| TGs/HDL-C | |  | |  | |  | |  | |  | |  |
|  | T1 | |  | | <1.35 | | 117/688 | | 7136 | | 16.4(13.7-19.6) | |
|  | T2 | |  | | 1.35-2.39 | | 148/731 | | 7559 | | 19.6(16.7-23.0) | |
|  | T3 | |  | | ≥2.39 | | 193/861 | | 9170 | | 21.0(18.3-24.2) | |
|  | |  | |  | |  | |  | |  | |  |
| Women(N=331) | |  | |  | |  | |  | |  | |  |
| TC | |  | |  | |  | |  | |  | |  |
|  | T1 | |  | | <5.15 | | 39/751 | | 7917 | | 4.9(3.6-6.7) | |
|  | T2 | |  | | 5.15-6.14 | | 91/874 | | 9447 | | 9.6(7.8-11.8) | |
|  | T3 | |  | | ≥6.14 | | 201/1149 | | 12685 | | 15.8(13.8-18.2) | |
| LDL-C | |  | |  | |  | |  | |  | |  |
|  | T1 | |  | | <3.49 | | 42/810 | | 8545 | | 4.9(3.6-6.5) | |
|  | T2 | |  | | 3.49-4.32 | | 103/908 | | 9891 | | 10.4(8.6-12.6) | |
|  | T3 | |  | | ≥4.32 | | 186/1056 | | 11613 | | 16.0(13.9-18.5) | |
| HDL-C | |  | |  | |  | |  | |  | |  |
|  | T1 | |  | | <0.93 | | 74/679 | | 7314 | | 10.12(8.0-12.7) | |
|  | T2 | |  | | 0.93-1.18 | | 101/790 | | 8411 | | 12.0(9.9-14.6) | |
|  | T3 | |  | | ≥1.18 | | 156/1305 | | 14329 | | 10.9(9.3-12.7) | |
| Non-HDL-C | |  | |  | |  | |  | |  | |  |
|  | T1 | |  | | <4.09 | | 43/810 | | 8523 | | 5.0(3.7-6.8) | |
|  | T2 | |  | | 4.09-5.08 | | 98/917 | | 10018 | | 9.8(8.0-11.9) | |
|  | T3 | |  | | ≥5.08 | | 190/1047 | | 11507 | | 10.5(14.3-19.0) | |
| CVD: cardiovascular disease, TC: total cholesterol, LDL-C: low density lipoprotein cholesterol, HDL-C: high density lipoprotein cholesterol, TGs: triglycerides.  ^*^Per 1000 person-year. | | | | | | | | | | | |  |

| Supplemental Table S1 (continued) | | | | | | |
| --- | --- | --- | --- | --- | --- | --- |
|  | |  | **Lipid limits** | **Event/At risk** | **Person-year** | **CVD incidence ^*^**  **(95% CI)** |
| TGs | |  |  |  |  |  |
|  | T1 |  | <1.5 | 64/915 | 9868 | 6.8(5.3-8.6) |
|  | T2 |  | 1.5-2.33 | 105/942 | 101146 | 10.3(8.5-12.5) |
|  | T3 |  | ≥2.33 | 159/917 | 10035 | 15.8(13.7-18.5) |
| TC/HDL-C | |  |  |  |  |  |
|  | T1 |  | <4.75 | 80/1061 | 11353 | 7.0(5.6-8.8) |
|  | T2 |  | 4.75-6.12 | 113/906 | 9900 | 11.4(9.5-13.7) |
|  | T3 |  | ≥6.12 | 138/807 | 8796 | 15.7(13.3-18.5) |
| TGs/HDL-C | |  |  |  |  |  |
|  | T1 |  | <1.35 | 88/1027 | 11126 | 7.9(6.4-9.7) |
|  | T2 |  | 1.35-2.39 | 121/950 | 10210 | 11.8(9.9-14.2) |
|  | T3 |  | ≥2.39 | 122/797 | 8713 | 14.0(11.7-16.7) |
|  | |  |  |  |  |  |
| Total (N=789) | |  |  |  |  |  |
| TC | |  |  |  |  |  |
|  | T1 |  | <5.15 | 184/1716 | 17661 | 10.4(9.0-12.0) |
|  | T2 |  | 5.15-6.14 | 247/1648 | 17762 | 13.9(12.3-15.7) |
|  | T3 |  | ≥6.14 | 358/1690 | 18490 | 19.4(17.4-21.5) |
| LDL-C | |  |  |  |  |  |
|  | T1 |  | <3.49 | 177/1729 | 17828 | 9.9(8.7-11.5) |
|  | T2 |  | 3.49-4.32 | 262/1674 | 18108 | 14.5(12.8-16.3) |
|  | T3 |  | ≥4.32 | 350/1651 | 17978 | 19.5(17.5-21.6) |
| HDL-C | |  |  |  |  |  |
|  | T1 |  | <0.93 | 302/1801 | 19005 | 15.9(14.2-17.8) |
|  | T2 |  | 0.93-1.19 | 225/1420 | 14993 | 15.0(13.2-17.1) |
|  | T3 |  | ≥1.19 | 262/833 | 19915 | 13.1(11.6-14.8) |
| Non-HDL-C | |  |  |  |  |  |
|  | T1 |  | <4.09 | 178/1709 | 17609 | 10.1(8.7-11.7) |
|  | T2 |  | 4.09-5.08 | 257/1690 | 18305 | 14.0(12.4-18.9) |
|  | T3 |  | ≥5.08 | 354/1655 | 17999 | 19.7(17.7-21.8) |
| TGs | |  |  |  |  |  |
|  | T1 |  | <1.5 | 205/1712 | 17946 | 11.4(9.9-13.1) |
|  | T2 |  | 1.5-2.33 | 249/1665 | 17762 | 14.0(12.4-15.9) |
|  | T3 |  | ≥2.33 | 335/1677 | 18206 | 18.4(16.5-20.5) |
| TC/HDL-C | |  |  |  |  |  |
|  | T1 |  | <4.75 | 183/1723 | 18114 | 10.1(8.7-11.7) |
|  | T2 |  | 4.75-6.12 | 266/1677 | 18051 | 14.7(13.1-16.6) |
|  | T3 |  | ≥6.12 | 340/1654 | 17748 | 19.1(17.2-21.3) |
| TGs/HDL-C | |  |  |  |  |  |
|  | T1 |  | <1.35 | 205/1715 | 18262 | 11.2(9.8-12.9) |
|  | T2 |  | 1.35-2.39 | 269/1681 | 17768 | 15.1(13.4-17.1) |
|  | T3 |  | ≥2.39 | 315/1658 | 17883 | 17.6(15.8-19.7) |
| CVD: cardiovascular disease, TC: total cholesterol, LDL-C: low density lipoprotein cholesterol, HDL-C: high density lipoprotein cholesterol, TGs: triglycerides.  ^*^Per 1000 person-year. | | | | | | |

| Supplemental Table S2 cardiovascular mortality rate (per 1000 person-year) of different serum lipid markers and lipid indices among total participants (n=5518); Tehran Lipid and Glucose Study (TLGS) (2001-2012). | | | | | | |
| --- | --- | --- | --- | --- | --- | --- |
|  | |  | **Lipid limits** | **Event/At risk** | **Person-year** | **Mortality rate^*^**  **(95% CI)** |
| CV mortality (n=279) | | |  |  |  |  |
| TC | |  |  |  |  |  |
|  | T1 |  | <5.15 | 71/1831 | 18709 | 3.8(3.0-4.8) |
|  | T2 |  | 5.15-6.14 | 92/1807 | 19297 | 4.8(3.9-5.8) |
|  | T3 |  | ≥6.14 | 116/1880 | 20391 | 5.7(4.7-6.8) |
| LDL-C | |  |  |  |  |  |
|  | T1 |  | <3.49 | 72/1840 | 18843 | 3.8(3.0-4.8) |
|  | T2 |  | 3.49-4.32 | 94/1839 | 19707 | 4.8(3.9-5.8) |
|  | T3 |  | ≥4.32 | 113/1839 | 19847 | 5.7(4.7-6.8) |
| HDL-C | |  |  |  |  |  |
|  | T1 |  | <0.93 | 106/1980 | 20676 | 5.1(4.2-6.2) |
|  | T2 |  | 0.93-1.19 | 73/1558 | 16341 | 4.5(3.5-5.6) |
|  | T3 |  | ≥1.19 | 100/1980 | 21380 | 4.7(3.8-5.7) |
| Non-HDL-C | |  |  |  |  |  |
|  | T1 |  | <4.09 | 73/1824 | 18674 | 3.9(3.1-4.9) |
|  | T2 |  | 4.09-5.08 | 97/1855 | 19887 | 4.9(4.0-5.9) |
|  | T3 |  | ≥5.08 | 109/1839 | 19835 | 5.5(4.5-6.6) |
| TGs | |  |  |  |  |  |
|  | T1 |  | <1.5 | 98/1838 | 19174 | 5.1(4.2-6.2) |
|  | T2 |  | 1.5-2.33 | 84/1822 | 19254 | 4.4(3.5-5.4) |
|  | T3 |  | ≥2.33 | 97/1858 | 19968 | 4.8(4.0-5.9) |
| TC/ HDL-C | |  |  |  |  |  |
|  | T1 |  | <4.75 | 85/1842 | 19258 | 4.4(3.6-5.4) |
|  | T2 |  | 4.75-6.12 | 87/1834 | 19552 | 4.4(3.6-5.5) |
|  | T3 |  | ≥6.12 | 107/1842 | 19587 | 5.5(4.5-6.6) |
| TGs/ HDL-C | |  |  |  |  |  |
|  | T1 |  | <1.35 | 89/1840 | 19499 | 4.6(3.7-5.6) |
|  | T2 |  | 1.35-2.39 | 105/1839 | 19257 | 5.4(4.5-5.6) |
|  | T3 |  | ≥2.39 | 85/1839 | 19641 | 4.3(3.5-5.3) |
| CV: cardiovascular, TC: total cholesterol, LDL-C: low density lipoprotein cholesterol, HDL-C: high density lipoprotein cholesterol, TGs: triglycerides.  ^*^Per 1000 person-year. | | | | | | |

| Supplemental Table S3 Non-cardiovascular mortality rate (per 1000 person-year) of different serum lipid markers and lipid indices among total participants (n=5518); Tehran Lipid and Glucose Study (TLGS) (2001-2012). | | | | | | | |
| --- | --- | --- | --- | --- | --- | --- | --- |
|  | |  | **Lipid limits** | **Event/At risk** | **Person-year** | **Mortality rate^*^**  **(95% CI)** | |
| Non-CV mortality (n=270) | | | |  |  |  | |
| TC | |  |  |  |  |  | |
|  | T1 |  | <5.15 | 106/1831 | 18709 | 5.5(4.7-6.8) | |
|  | T2 |  | 5.15-6.14 | 82/1807 | 19297 | 4.2(3.4-5.3) | |
|  | T3 |  | ≥6.14 | 82/1880 | 20391 | 4.0(3.2-5.0) | |
| LDL-C | |  |  |  |  |  | |
|  | T1 |  | <3.49 | 112/1840 | 18843 | 5.9(4.9-7.2) | |
|  | T2 |  | 3.49-4.32 | 74/1839 | 19707 | 3.7(3.0-4.7) | |
|  | T3 |  | ≥4.32 | 84/1839 | 19847 | 4.2(3.4-5.2) | |
| HDL-C | |  |  |  |  |  | |
|  | T1 |  | <0.93 | 94/1980 | 20676 | 4.5(3.7-5.6) | |
|  | T2 |  | 0.93-1.19 | 66/1558 | 16341 | 4.0(3.2-5.1) | |
|  | T3 |  | ≥1.19 | 110/1980 | 21380 | 5.1(4.3-6.2) | |
| Non-HDL-C | |  |  |  |  |  | |
|  | T1 |  | <4.09 | 113/1824 | 18674 | 6.1(5.0-7.3) | |
|  | T2 |  | 4.09-5.08 | 73/1855 | 19887 | 3.7(2.9-4.6) | |
|  | T3 |  | ≥5.08 | 84/1839 | 19835 | 4.2(3.4-5.2) | |
| TGs | |  |  |  |  |  | |
|  | T1 |  | <1.5 | 111/1838 | 19174 | 5.8(4.8-7.0) | |
|  | T2 |  | 1.5-2.33 | 89/1822 | 19254 | 4.6(3.8-5.7) | |
|  | T3 |  | ≥2.33 | 70/1858 | 19968 | 3.5(2.8-4.4) | |
| TC/HDL-C | |  |  |  |  |  | |
|  | T1 |  | <4.75 | 109/1842 | 19258 | 5.7(4.7-6.8) | |
|  | T2 |  | 4.75-6.12 | 89/1834 | 19552 | 4.6(3.7-5.6) | |
|  | T3 |  | ≥6.12 | 72/1842 | 19587 | 3.7(2.9-4.6) | |
| TGs/HDL-C | |  |  |  |  |  | |
|  | T1 |  | <1.35 | 107/1840 | 19499 | 5.5(4.5-6.6) | |
|  | T2 |  | 1.35-2.39 | 92/1839 | 19257 | 4.8(3.9-5.9) | |
|  | T3 |  | ≥2.39 | 71/1839 | 19641 | 3.6(2.9-5.7) | |
| CV: cardiovascular, TC: total cholesterol, LDL-C: low density lipoprotein cholesterol, HDL-C: high density lipoprotein cholesterol, TGs: triglycerides.  ^*^Per 1000 person-year. | | | | | | |  |

| Supplemental Table S4 Hazard ratios of lipid measures for predicting first cardiovascular disease events among participants without prevalent CVD in those with available data on HOMA-IR (n=1548); Tehran Lipid and Glucose Study (TLGS) (2001-2012)^*^. | | | | | | | |  |
| --- | --- | --- | --- | --- | --- | --- | --- | --- |
|  |  | **Model 1** | | |  | **Model 2** | |  |
|  |  | **SD (mmol/L)** | **HR (95% CI)** | **p-value** |  | **HR (95% CI)** | **p-value** |  |
| TC |  | 1.18 | 1.15(1.0-1.32) | 0.05 |  | 1.07(0.92-1.23) | 0.39 |  |
| LDL-C |  | 1.0 | 1.16(1.01-1.33) | 0.03 |  | 1.08(0.94-1.25) | 0.28 |  |
| HDL-C |  | 0.28 | 0.85(0.73-1.0) | 0.05 |  | 0.91(0.77-1.07) | 0.27 |  |
| Non-HDL-C |  | 1.17 | 1.18(1.03-1.35) | 0.01 |  | 1.05(0.91-1.21) | 0.49 |  |
| ln-TGs |  | 0.2 | 1.21(1.05-1.39) | 0.008 |  | 1.02(0.87-1.19) | 0.84 |  |
| TC/HDL-C |  | 1.64 | 1.20(1.05-1.38) | 0.007 |  | 1.13(0.97-1.32) | 0.12 |  |
| ln-TGs/HDL-C |  | 0.46 | 1.22(1.06-1.41) | 0.006 |  | 1.04(0.89-1.22) | 0.6 |  |
| Model 1: lipid profile + gender; model 2= model 1 + blood pressure status (i.e. normotension, prehypertension and hypertension status), glucose tolerance status (normal glucose tolerance, prediabetes and diabetes), education status, low physical activity, current smoker, lipid lowering drugs, body mass index and HOMA-IR.  *Hazard ratios (HR) indicate the increase risk for a 1-SD increase of each lipid parameter.  CVD: cardiovascular disease, HOMA-IR: homeostatic model assessment-insulin resistance, SD: standard deviation, TC: total cholesterol, LDL-C: low density lipoprotein cholesterol, HDL-C: high density lipoprotein cholesterol, ln-TGs: logarithm-transformed triglycerides, CI: confidence interval. | | | | | | | |  |

| Supplemental Table S5 Hazard ratios of lipid measures for predicting first cardiovascular disease events among participants without prevalent CVD based on lipid profile tertiles CVD in those with available data on HOMA-IR (n=1548); Tehran Lipid and Glucose Study (TLGS) (2001-2012). | | | | | | | | | | |
| --- | --- | --- | --- | --- | --- | --- | --- | --- | --- | --- |
|  |  | **Model 1** | | |  |  | **Model 2** | | |  |
|  |  | **Tertiles of variables** | | | **P _for trend*_** |  | **Tertiles of variables** | | | **P _for trend*_** |
|  |  | **1** | **2** | **3** |  |  | **1** | **2** | **3** |  |
| TC, mmol/L |  | Reference | 1.55(1.06-2.27) | 1.71(1.16-2.51) | 0.007 |  | Reference | 1.56(1.05-2.34) | 1.57(1.05-2.36) | 0.037 |
| LDL-C, mmol/L |  | Reference | 1.59(1.09-2.32) | 1.73(1.18-2.54) | 0.006 |  | Reference | 1.65(1.11-2.47) | 1.66(1.10-2.50) | 0.02 |
| HDL-C, mmol/L |  | Reference | 0.89(0.63-1.27) | 0.73(0.51-1.03) | 0.07 |  | Reference | 0.92(0.64-1.31) | 0.83(0.57-1.20) | 0.33 |
| Non-HDL-C |  | Reference | 1.61(1.10-2.34) | 1.73(1.18-2.53) | 0.007 |  | Reference | 1.61(1.08-2.40) | 1.58(1.05-2.39) | 0.04 |
| TGs, mmol/L |  | Reference | 1.15(0.79-1.68) | 1.62(1.14-2.30) | 0.006 |  | Reference | 0.95(0.65-1.39) | 1.10(0.75-1.62) | 0.57 |
| TC/HDL-C |  | Reference | 1.39(0.96-2.02) | 1.81(1.26-2.59) | 0.001 |  | Reference | 1.28(0.87-1.89) | 1.48(1.01-2.17) | 0.05 |
| TGs/HDL-C |  | Reference | 1.18(0.82-1.69) | 1.63(1.15-2.31) | 0.006 |  | Reference | 0.99(0.67-1.44) | 1.16(0.79-1.71) | 0.41 |
| Model 1: lipid profile tertiles + gender; model 2= model 1 + blood pressure status (i.e. normotension, prehypertension and hypertension status), glucose tolerance status (normal glucose tolerance, prediabetes and diabetes), education status, low physical activity, current smoker, lipid lowering drugs, body mass index and HOMA-IR.  CVD: cardiovascular disease, HOMA-IR: homeostatic model assessment-insulin resistance, TC: total cholesterol, LDL-C: low density lipoprotein cholesterol, HDL-C: high density lipoprotein cholesterol, TGs: triglycerides, CI: confidence interval.  ^*^P-values were calculated using age scale Cox proportional hazards regression models | | | | | | | | | | |
